# Supplementary material for: Social media usage reveals recovery of small businesses after natural hazard events
Source: Nat Commun. 2020 Apr 2;11:1629. doi: 10.1038/s41467-020-15405-7 (PMC7118130; doi:10.1038/s41467-020-15405-7)
Supplement: Supplementary file 1 — Supplementary Information [file 41467_2020_15405_MOESM1_ESM.pdf]

Social media usage reveals small businesses recovery after natural hazard events: Supplementary Materials

Robert Eyre[1], Flavia De Luca[2,*], and Filippo Simini[1,3,†]

[1]University of Bristol, Department of Engineering Mathematics, Bristol, BS8 1UB, UK
[2]University of Bristol, Department of Civil Engineering, Bristol, BS8 1UB, UK
[3]The Alan Turing Institute, London, UK
[*]To whom correspondence should be addressed; E-mail: flavia.deluca@bristol.ac.uk
[†]To whom correspondence should be addressed; E-mail: f.simini@bristol.ac.uk

Supplementary Figures

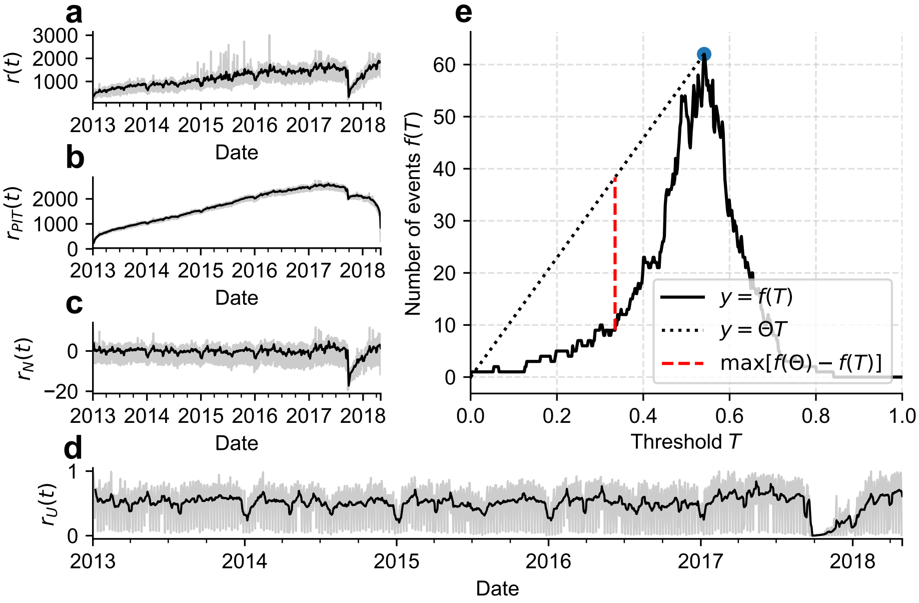

Supplementary Figure 1: Steps of the methodology to estimate the downtime of small businesses applied to San Juan, Puerto Rico. **(a)** Time series of the total number of posts of all businesses ($r(t)$), shown with weekly rolling mean (black solid line). **(b)** Step 1, single business Probability Integral Transformed data (p_{PIT}) **(c)** Step 2, data is shifted and rescaled according the number of active businesses **(d)** Step 4, PIT applied on the aggregated and transformed time series ($r_N(t)$) **(e)** Downtime detection using the 'elbow method'.

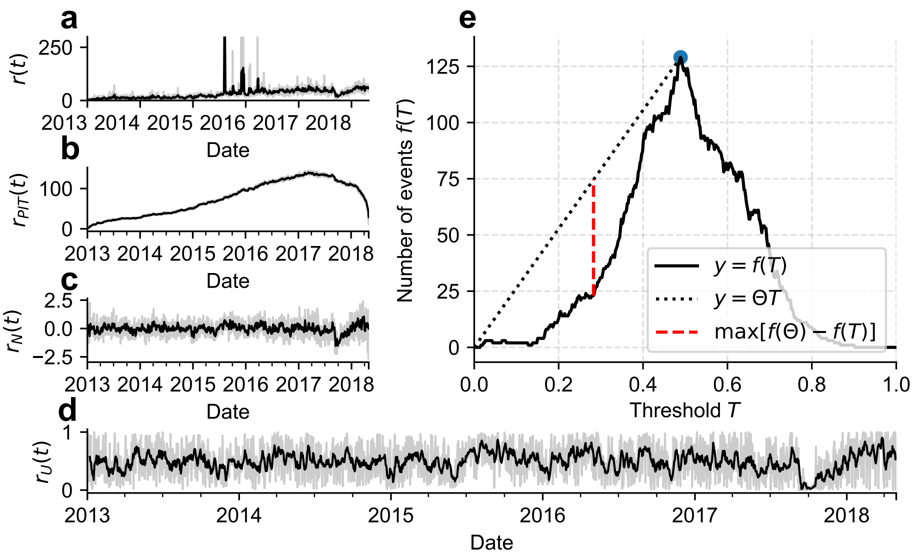

Supplementary Figure 2: Steps of the methodology to estimate the downtime of small businesses applied to Juchitán de Zaragoza, Mexico. **(a)** Time series of the total number of posts of all businesses ($r(t)$), shown with weekly rolling mean (black solid line). **(b)** Step 1, single business Probability Integral Transformed data (p_{PIT}) **(c)** Step 2, data is shifted and rescaled according the number of active businesses **(d)** Step 4, PIT applied on the aggregated and transformed time series ($r_N(t)$) **(e)** Downtime detection using the 'elbow method'.

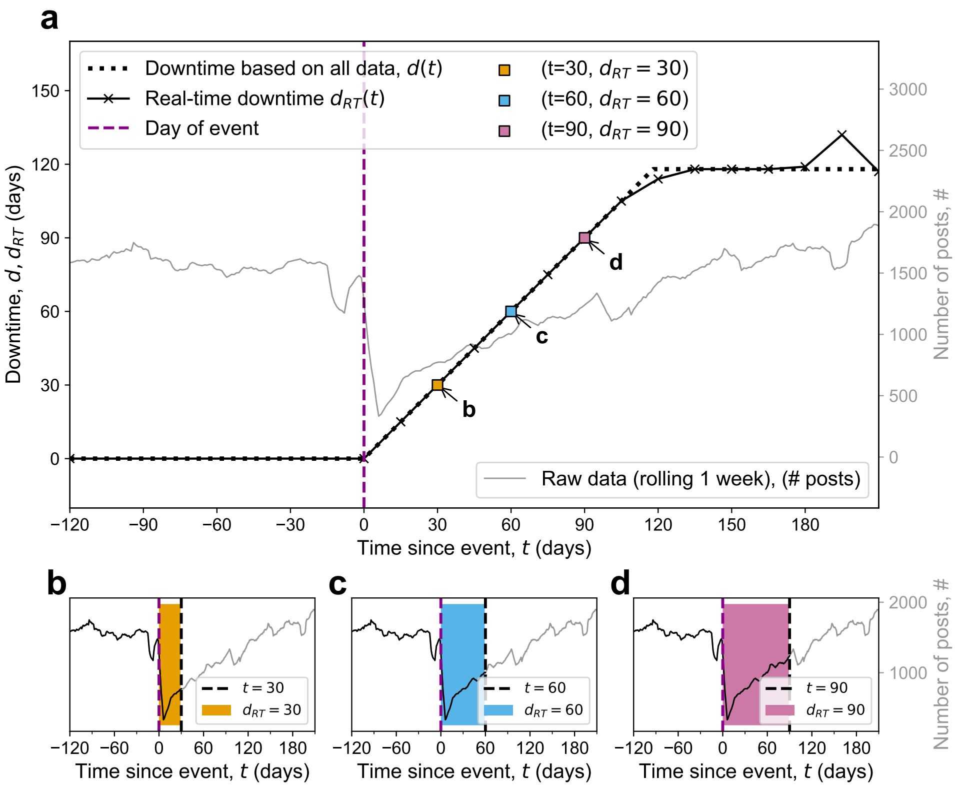

Supplementary Figure 3: Downtime detection in real-time ($d_{RT}(t)$) for San Juan, Puerto Rico. **(a)** Data are cropped at regular intervals to simulate real-time data collection (crossed markers along solid line). Square markers at $(t, d_{RT}(t))$ indicate the real time estimates for $t = 30$, 60, 90 days. Dotted line indicates ideal downtime, $d(t)$, estimated using all data. **(b-d)** Black solid lines denote the cropped data used to estimate downtime at $t = 30$, 60, 90 days after the event (dashed magenta line) respectively. The cutoff t is shown with a black dashed line. The estimated downtimes $d_{RT}(t)$ are shown with coloured areas.

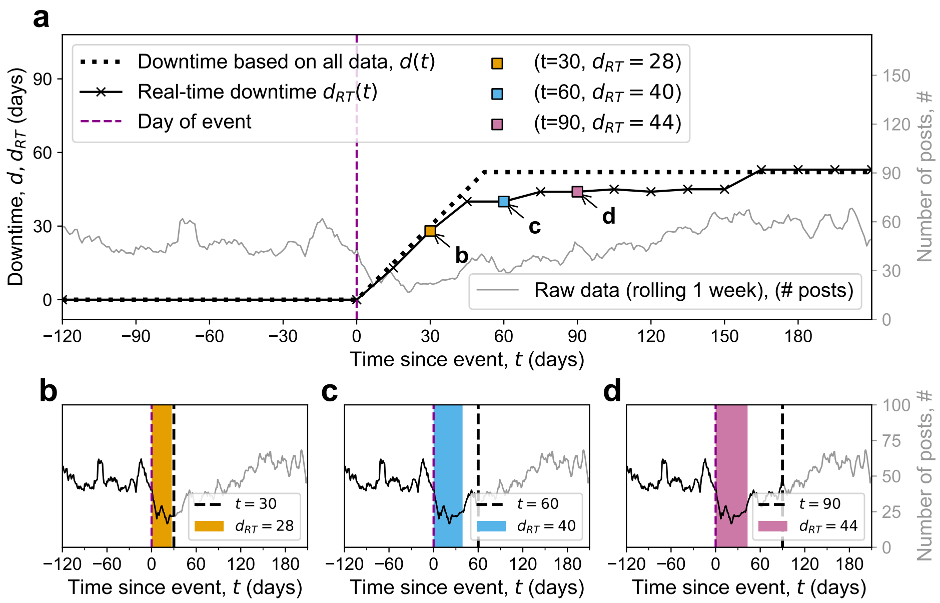

Supplementary Figure 4: Downtime detection in real-time ($d_{RT}(t)$) for Juchitán de Zaragoza, Mexico. **(a)** Data are cropped at regular intervals to simulate real-time data collection (crossed markers along solid line). Square markers at $(t, d_{RT}(t))$ indicate the real time estimates for $t = 30$, 60, 90 days. Dotted line indicates ideal downtime, $d(t)$, estimated using all data. **(b-d)** Black solid lines denote the cropped data used to estimate downtime at $t = 30$, 60, 90 days after the event (dashed magenta line) respectively. The cutoff t is shown with a black dashed line. The estimated downtimes $d_{RT}(t)$ are shown with coloured areas.

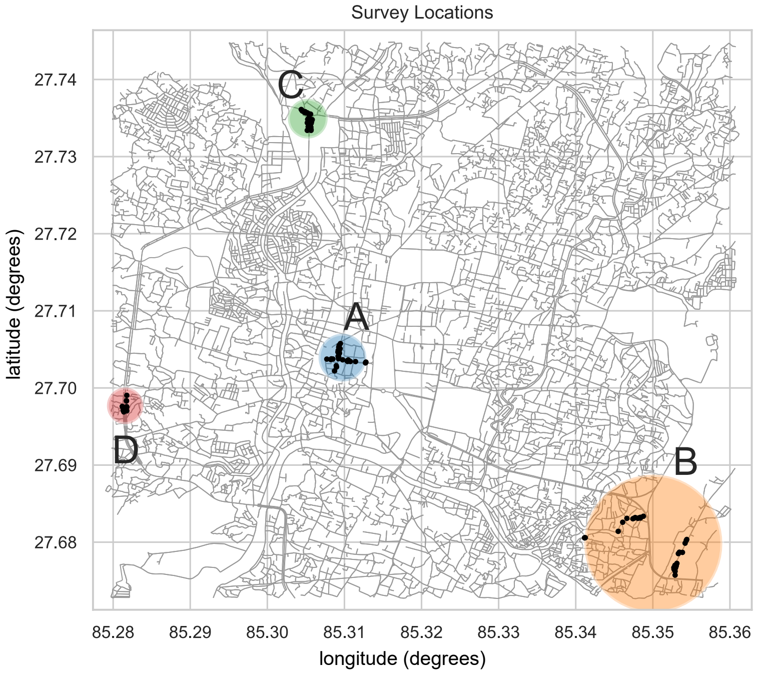

Supplementary Figure 5: Survey locations in the study by De Luca et al. [1]. Road networks collected from OpenStreetMap [2] using OSMnx [3]. OpenStreetMap data is available under the Open Database License and licensed as CC BY-SA `https://creativecommons.org/licenses/by-sa/2.0/`.

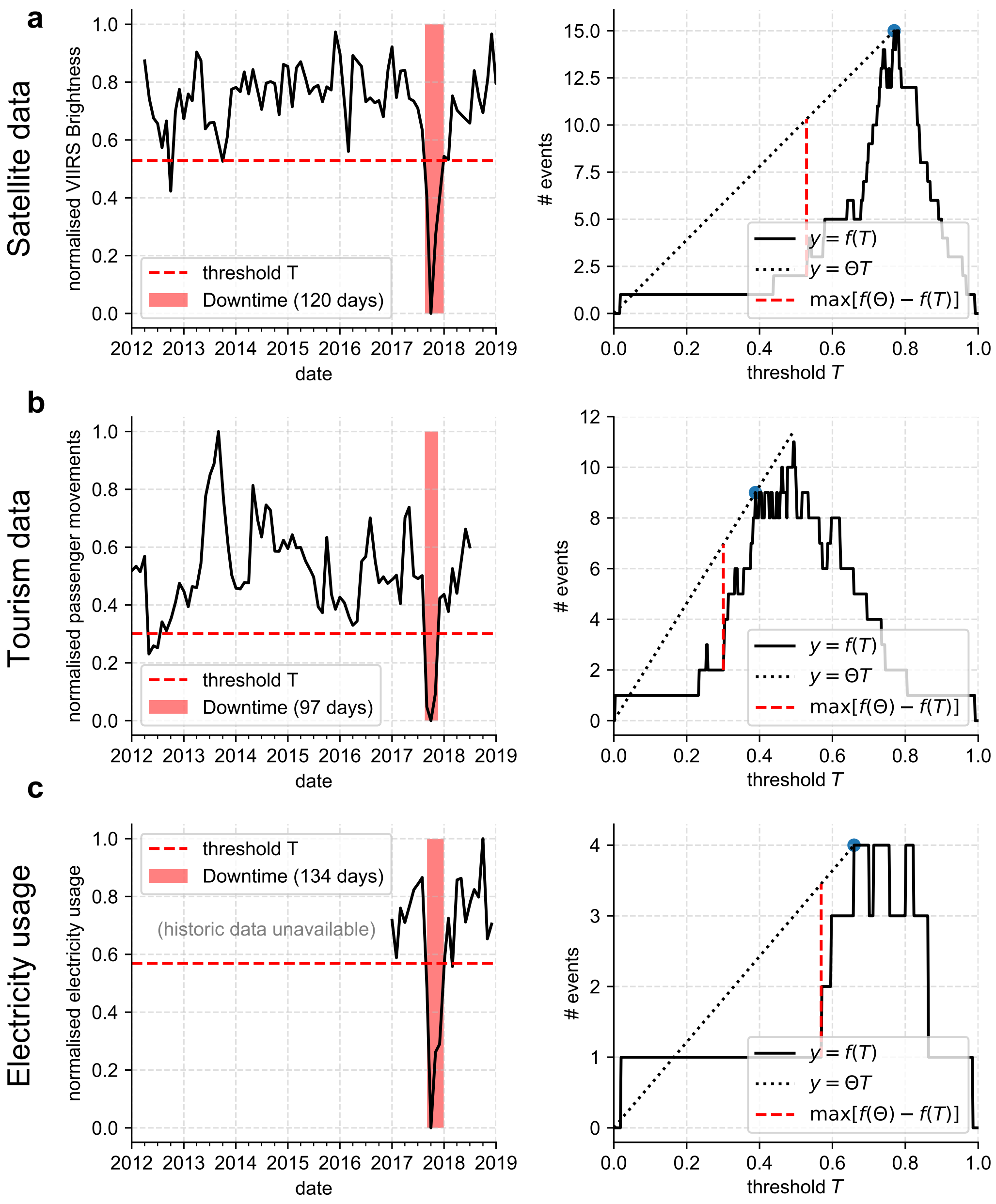

Supplementary Figure 6: Normalised data used to validate the estimated downtime in San Juan, Puerto Rico. Data was normalised by rescaling the values so they fall between 0 and 1, and then the elbow method was used to determine downtime. (a) Satellite data looking at average brightness provided by [4] (b) Tourism data containing statistics on incoming tourists to the Port of Old San Juan (c) Bought electricity provided by the American government.

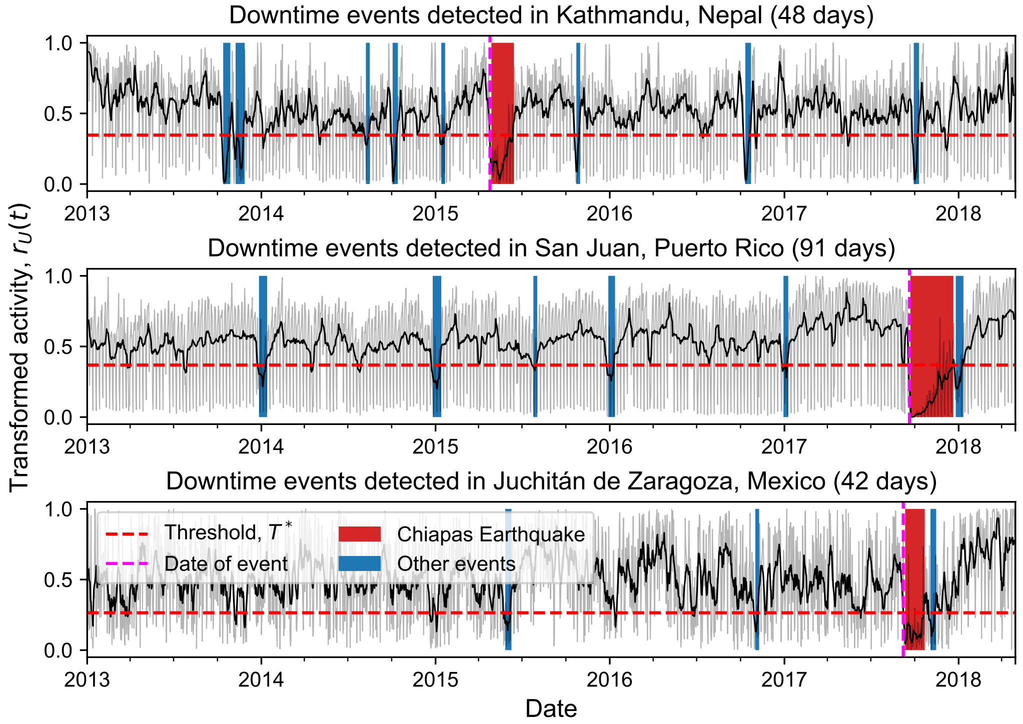

Supplementary Figure 7: Downtime considering businesses just posting one year before the event date. Natural hazard event is shown in red, with other detected events shown in blue. The date of the event has been highlighted in magenta.

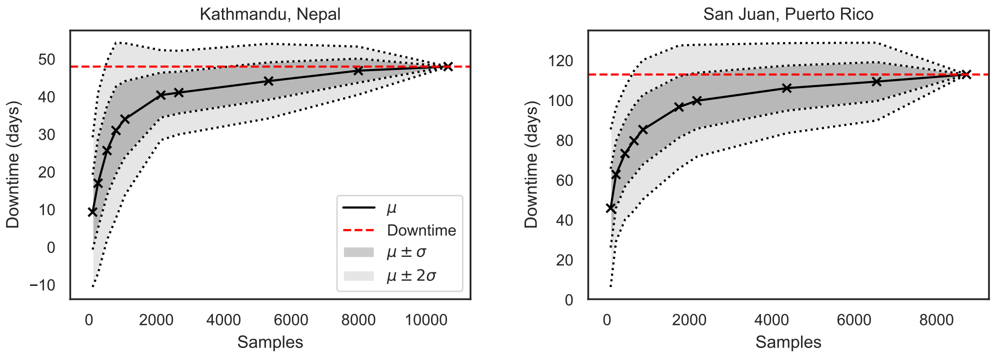

Supplementary Figure 8: Business samples of different sizes are taken in Kathmandu, Nepal (left) and San Juan, Puerto Rico (right). Downtimes are calculated, and then the process is repeated. Average downtime shown with one and two standard deviations from the mean.

Supplementary Tables

Start date	End date	Duration (days)	Event
2013-10-13	2013-10-29	17	Dashain 2013 (5th Oct - 17th Oct)
2013-11-07	2013-11-27	21	Tihar 2013 (1st Nov - 5th Nov)
2014-10-03	2014-10-14	12	Dashain 2014 (24th Sep - 7th Oct)
2015-04-28	2015-06-14	48	**Gorkha Earthquake (25th April)**
2015-10-22	2015-11-01	11	Dashain 2015 (13th Oct - 26th Oct)
2016-10-10	2016-10-25	16	Dashain 2016 (1st Oct - 15th Oct)
2017-05-04	2017-05-21	18	Unknown
2017-09-28	2017-10-14	17	Dashain 2017 (20th Sep - 4th Oct)
2017-10-24	2017-11-05	13	Unknown
2017-11-26	2017-12-18	23	Unknown
2018-02-04	2018-02-11	8	Unknown

Supplementary Table 1: Detected events in Kathmandu, Nepal.

Start date	End date	Duration (days)	Event
2013-12-30	2014-01-10	12	Christmas / New Year 2013
2014-12-27	2015-01-12	17	Christmas / New Year 2014
2015-12-29	2016-01-12	15	Christmas / New Year 2015
2016-12-30	2017-01-09	11	Christmas / New Year 2016
2017-09-21	2018-01-16	118	**Hurricane Maria (20th Sep)**

Supplementary Table 2: Detected events in San Juan, Puerto Rico.

Start date	End date	Duration (days)	Event
2015-05-26	2015-06-09	15	Unknown
2016-11-01	2016-11-08	8	All Saints' Day / Day of the Dead (1/2 Nov)
2017-09-10	2017-10-31	52	**M8.2 Chiapas Earthquake (7th Sep)**
2017-11-02	2017-11-13	12	All Saints' Day / Day of the Dead (1/2 Nov)
2017-11-21	2017-11-30	10	Revolution Day (20th Nov)

Supplementary Table 3: Detected events in Juchitán de Zaragoza, Mexico.

	Mean downtime (days)	Standard deviation (days)	Number of surveys
Area A	29	44	27
Area B	15	20	31
Area C	61	88	25
Area D	78	62	10
All regions	41	62	93

Supplementary Table 4: Downtime (days) reported in four areas in Kathmandu, Nepal, from the surveys taken by De Luca et al. [1]

# posts / Daily post rate	0	1/7	1/2	5/7	1
0	$48_{(10656)}$	$47_{(2467)}$	$47_{(1313)}$	$35_{(872)}$	$37_{(368)}$
100	$48_{(2504)}$	$47_{(989)}$	$46_{(517)}$	$35_{(291)}$	$50_{(158)}$
200	$47_{(1373)}$	$47_{(801)}$	$46_{(439)}$	$35_{(253)}$	$51_{(143)}$
300	$47_{(947)}$	$47_{(682)}$	$47_{(394)}$	$35_{(234)}$	$51_{(135)}$
400	$46_{(679)}$	$47_{(587)}$	$36_{(358)}$	$35_{(215)}$	$51_{(125)}$

Supplementary Table 5: Overall downtime reported over the whole of Kathmandu, Nepal, with different filters on the businesses (by the number of posts they have made, and the daily average posting rate). Number of businesses that meet the criterion are listed in brackets for each reported downtime.

# posts / Daily post rate	0	1/7	1/2	5/7	1
0	$118_{(8725)}$	$113_{(3313)}$	$112_{(2013)}$	$110_{(1352)}$	$111_{(700)}$
100	$119_{(3461)}$	$117_{(2022)}$	$113_{(1289)}$	$110_{(818)}$	$111_{(482)}$
200	$117_{(2411)}$	$117_{(1766)}$	$113_{(1180)}$	$110_{(760)}$	$111_{(455)}$
300	$117_{(1838)}$	$116_{(1547)}$	$112_{(1085)}$	$111_{(719)}$	$110_{(433)}$
400	$117_{(1512)}$	$113_{(1397)}$	$112_{(1021)}$	$110_{(687)}$	$107_{(421)}$

Supplementary Table 6: Overall downtime reported over the whole of San Juan, Puerto Rico with different filters on the businesses (by the number of posts they have made, and the daily average posting rate). Number of businesses that meet the criterion are listed in brackets for each reported downtime.

# posts / Daily post rate	0	1/7	1/2	5/7	1
0	$52_{(573)}$	$42_{(200)}$	$46_{(104)}$	$37_{(73)}$	$43_{(32)}$
100	$42_{(126)}$	$45_{(66)}$	$48_{(34)}$	$46_{(23}$	$6_{(15)}$
200	$44_{(72)}$	$45_{(56)}$	$46_{(30)}$	$45_{(20)}$	$43_{(13)}$
300	$45_{(48)}$	$71_{(41)}$	$46_{(25)}$	$43_{(16)}$	$43_{(11)}$
400	$9_{(33)}$	$8_{(30)}$	$9_{(23)}$	$12_{(15)}$	$43_{(11)}$

Supplementary Table 7: Overall downtime reported over the whole of Juchitán de Zaragoza, Mexico, with different filters on the businesses (by the number of posts they have made, and the daily average posting rate). Number of businesses that meet the criterion are listed in brackets for each reported downtime.

Supplementary Notes

Supplementary Note 1

To validate the automatic text analysis approach described in Section 3.1, we manually read 19,928 posts from businesses in Kathmandu and estimated the reopening date of each business based on the context of all of its posts. We obtained a downtime of 50 days, which is compatible with the 51 days of the automatic text analysis algorithm.

The application of the text analysis approach used in validating downtime emphasised some of the limitations of such kind of analyses, as discussed in [5]. We list these limitations below:

- **Ambiguity about recovery status**. We found cases where businesses state that they will reopen, and then never post again. It is unclear here whether they actually recovered.
- **Businesses do not post whether they have reopened**. The majority of businesses do not explicitly state that they are open - where we have collected 40946 posts in Kathmandu, 94611 posts in San Juan and 4536 posts in Juchitán de Zaragoza respectively, there were only hundred of posts containing the keywords that were used to filter the messages.
- **Keywords are difficult to establish.** Keywords are specific to each region. Local dialects and slang make the task of identifying relevant keywords difficult when validating this data using Facebook posts.
- **Repeated posts about recovery status**. Additionally, businesses who do say they have reopened often repeatedly posts that they have reopened. To deal with this case only the first posts to mention a keyword for each businesses is used for analysis.

Supplementary Note 2

In our analysis we consider all businesses that posted at least once, irrespective of the date of the event, hence we also include new businesses that started to post after the disaster. The rationale behind this choice is to use the same methodology to transform the entire time series, without differentiating between periods before and after the disaster. Computing the downtime considering only businesses posting since at least one year before the date of the event may produce more robust results because businesses with a long posting history have more data and better statistics. A possible downside of this approach is that downtime estimates could be less accurate because of the fewer businesses considered. Applying our methodology just to businesses that posted one year before the events in the three regions, we get downtime estimates similar to those obtained looking at all businesses. In particular, the number of businesses remaining after the filtering is 2,781 in Kathmandu, 6,616 in San Juan and 380 in Juchitán de Zaragoza. The estimated lengths of downtime after the natural disasters are 48 days in Kathmandu, 91 days in San Juan, and 42 days in Juchitán de Zaragoza. The estimate for Kathmandu is the same as the estimate using all the businesses, while the estimated downtimes are shorter for San Juan and Juchitán de Zaragoza. As shown in Supplementary Figure 6, a reduction of the downtime is expected when fewer businesses are considered. Note that in San Juan we detect an additional downtime of 17 days during the Christmas/New Year period, only 6 days after the end of the downtime due to Hurricane Maria. Combining these two downtimes we obtain an overall length of 114 days, which is very close to the 118 days estimated using all businesses.

Supplementary References

[1] De Luca, F. *et al.* Traffic data as proxy of business downtime after natural disasters: The case of kathmandu. In *11th National Conference on Earthquake Engineering* (2018).

[2] OpenStreetMap contributors. Planet dump retrieved from https://planet.osm.org . `https://www.openstreetmap.org` (2018).

[3] Boeing, G. Osmnx: New methods for acquiring, constructing, analyzing, and visualizing complex street networks. *Computers, Environment and Urban Systems* **65**, 126–139 (2017). URL `http://dx.doi.org/10.1016/j.compenvurbsys.2017.05.004`.

[4] Shermeyer, J. Assessment of electrical and infrastructure recovery in puerto rico following hurricane maria using a multisource time series of satellite imagery. In Michel, U. & Schulz, K. (eds.) *Earth Resources and Environmental Remote Sensing/GIS Applications IX* (SPIE, 2018). URL `http://dx.doi.org/10.1117/12.2325585`.

[5] Palen, L. & Anderson, K. M. Crisis informatics—new data for extraordinary times. *Science* **353**, 224–225 (2016). URL `https://science.sciencemag.org/content/353/6296/224`. `https://science.sciencemag.org/content/353/6296/224.full.pdf`.
